# Aspirin reduces the mortality risk of patients with community-acquired pneumonia: a retrospective propensity-matched analysis of the MIMIC-IV database

**DOI:** 10.3389/fphar.2024.1402386

**Published:** 2024-09-13

**Authors:** Guangdong Wang, Jiaolin Sun, Yaxin Zhang, Na Wang, Tingting Liu, Wenwen Ji, Lin Lv, Xiaohui Yu, Xue Cheng, Mengchong Li, Tinghua Hu, Zhihong Shi

**Affiliations:** ^1^ Department of Respiratory and Critical Care Medicine, First Affiliated Hospital of Xi’an Jiaotong University, Xi’an, Shanxi, China; ^2^ Department of Respiratory and Critical Care Medicine, Shanxi Provincial People’s Hospital, Xi’an, Shanxi, China; ^3^ Department of Neurology, Fujian Medical University Affiliated Xiamen Hong ’ai Hospital, Xiamen Fujian, China

**Keywords:** community-acquired pneumonia, intensive care unit, aspirin, MIMIC-IV database, mortality

## Abstract

**Background:**

Community-acquired pneumonia (CAP) is a common infectious disease characterized by inflammation of the lung parenchyma in individuals who have not recently been hospitalized. It remains a significant cause of morbidity and mortality worldwide. Aspirin is a widely used drug, often administered to CAP patients. However, the benefits of aspirin remain controversial.

**Objective:**

We sought to determine whether aspirin treatment has a protective effect on the outcomes of CAP patients.

**Methods:**

We selected patients with CAP from the Medical Information Mart for Intensive Care IV (MIMIC-IV) database. Propensity score matching (PSM) balanced baseline differences. A multivariate Cox regression model assessed the relationship between aspirin treatment and 28-day mortality.

**Results:**

A total of 3,595 patients were included, with 2,261 receiving aspirin and 1,334 not. After PSM, 1,219 pairs were matched. The 28-day mortality rate for aspirin users was 20.46%, lower than non-users. Multivariate Cox regression indicated aspirin use was associated with decreased 28-day mortality (HR 0.75, 95% CI 0.63–0.88, *p* < 0.001). No significant differences were found between 325 mg/day and 81 mg/day aspirin treatments in terms of 28-day mortality, hospital mortality, 90-day mortality, gastrointestinal hemorrhage, and thrombocytopenia. However, intensive care unit (ICU) stay was longer for the 325 mg/day group compared to the 81 mg/day group (4.22 vs. 3.57 days, *p* = 0.031).

**Conclusion:**

Aspirin is associated with reduced 28-day mortality in CAP patients. However, 325 mg/day aspirin does not provide extra benefits over 81 mg/day and may lead to longer ICU stays.

## 1 Introduction

Community-acquired pneumonia (CAP) refers to pulmonary parenchymal infectious inflammation that occurs outside the hospital, including pneumonia that develops after admission due to infection acquired outside the hospital. CAP is a common cause of intensive care unit (ICU) stay and a major cause of infectious disease mortality (Aliberti et al., 2016). In developing countries, the incidence rate of CAP ranges from 20% to 30%, notably higher than the 3%–4% observed in developed countries (Zhang et al., 2019). The overall mortality rate for hospitalized CAP patients stands at 13%, whereas it surges to over 35% for patients with severe CAP (Ewig et al., 2009). The mortality rate of CAP is approximately 25%–50%,which poses a huge burden on families and society (Feldman and Anderson, 2016; Niederman and Torres, 2022).

Aspirin, chemically known as acetylsalicylic acid, is a widely used medication with a rich history of over a century in both clinical and over-the-counter settings (Desborough and Keeling, 2017). Its primary functions span from reducing pain, fever, and inflammation to playing a pivotal role in preventing blood clots, thus significantly reducing the risk of heart attacks and strokes (Antithrombotic Trialists’ Collaboration, 2002). The underlying mechanisms of aspirin’s action are multifaceted. At its core, aspirin inhibits the enzyme cyclooxygenase (COX), leading to a decrease in the synthesis of prostaglandins and thromboxanes (Vane, 1971). Prostaglandins are substances that mediate inflammation and are involved in the regulation of the fever response and sensation of pain (Vane, 1978). By reducing prostaglandin production, aspirin effectively alleviates inflammation and diminishes pain and fever.

Among the causes of poor survival of CAP, sepsis and early cardiovascular events, which complicate the clinical course of pneumonia, may play an important role (Violi et al., 2017; Bjarnason et al., 2018). Therefore, it has been suggested that drugs that regulate inflammation and coagulation to prevent sepsis and cardiovascular events, such as macrolides, steroids, aspirin, statins, or others, may improve the outcome of severe pneumonia (Corrales-Medina and Musher, 2011; Torres et al., 2015). Previous studies found aspirin treatment in elderly CAP patients is associated with reduced mortality, a lower incidence of pleural effusion, milder respiratory disorders, decreased Sequential Organ Failure Assessment (SOFA) scores, and reduced rates of severe sepsis (Falcone et al., 2015). A long-term nationwide study enrolled 815 bacteremic pneumococcal pneumonia patients in Iceland also suggested that aspirin is associated with improved 30-day survival (Rögnvaldsson et al., 2022). Furthermore, research has shown that patients with severe CAP benefit more from treatment combining macrolides and aspirin than from conventional antibiotic therapy alone (Falcone et al., 2019). While other researchers have failed to find a significant benefit of aspirin use. Chalmers et al. conducted a prospective observational study in CAP patients, revealing that aspirin therapy had no impact on tracheal intubation rates, pleural effusion incidence, or 30-day mortality (Chalmers et al., 2008). A retrospective study enrolled 278 CAP patients showed that low-dose aspirin neither reversed platelet activation nor reduced the incidence of myocardial infarction (12% vs. 10%; *p* = 0.649) (Cangemi et al., 2014).

Whether aspirin use is associated with lower mortality in CAP patients admitted to ICU remains controversial. Therefore, we designed this observational study to research the potential beneficial effect of aspirin use among CAP patients.

## 2 Materials and methods

### 2.1 Database introduction

This study was a retrospective cohort study, and all data in this study were obtained from the Medical Information Mart for Intensive Care IV (MIMIC-IV) database (Johnson et al., 2023). The MIMIC-IV database is an expansive, freely accessible dataset that serves as a vital resource for the global research community, focusing on critical care. Developed by the Massachusetts Institute of Technology (MIT) in collaboration with the Beth Israel Deaconess Medical Center, MIMIC-IV encompasses detailed health data from over 40,000 patients who were admitted to critical care units between 2008 and 2019. This database includes a wide range of information, such as demographic details, vital signs, laboratory test results, medications, and diagnostic codes, providing a comprehensive view of patient care in the ICU. The first author, Guangdong Wang, who had completed the Collaborative Institutional Training Initiative (CITI) program and passed the “Conflicts of Interest” and “Data or Specimens Only Research” examinations (Certification ID: 60106105), was authorized to access the MIMIC-IV database.

### 2.2 Population selection criteria

**FIGURE 1:**
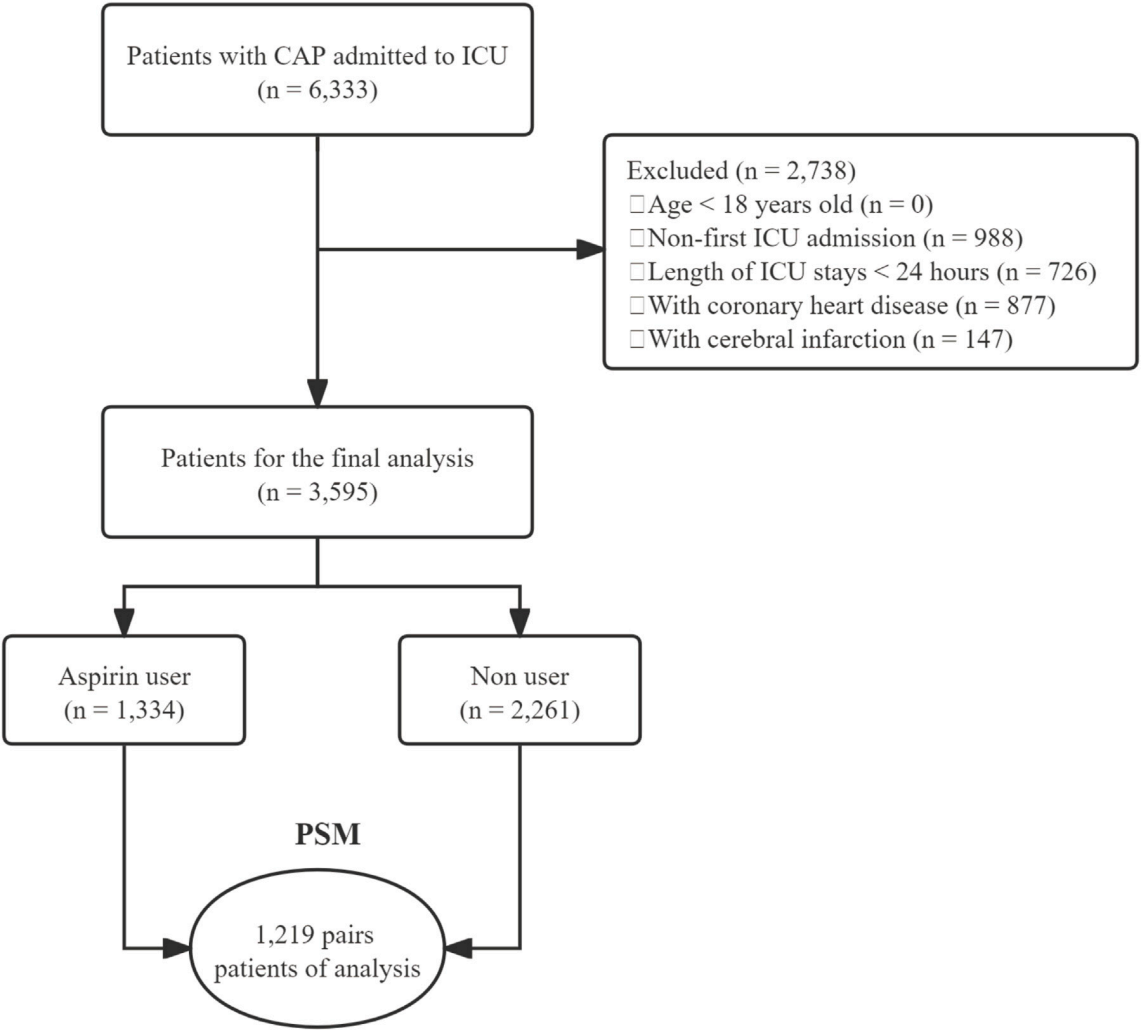
Flowchart of the study. CAP, community-acquired pneumonia; ICU, intensive care unit; PSM, propensity score matching.

The cohort study evaluated patients with CAP admitted to ICU, 6,333 CAP patients identified using International Classification of Diseases (ICD) codes. The initial cohort was refined by excluding 2,738 patients due to non-first ICU admissions, ICU stays shorter than 24 h, and pre-existing conditions such as coronary heart disease (877 patients) and cerebral infarction (147 patients). Exclusions for coronary heart disease and cerebral infarction were necessitated by the potential for long-term aspirin use prior to and continuing after admission, which could confound the assessment of aspirin’s therapeutic impact on CAP. Thus, to enhance the accuracy of evaluating aspirin’s role in CAP management, these patients were omitted to reduce bias associated with baseline aspirin consumption linked to chronic conditions. The final cohort comprised 3,595 patients, consisting of 1,334 aspirin users and 2,261 non-users. After propensity score matching (PSM), 1,219 pairs of patients were matched (Figure 1).

### 2.3 Aspirin exposure

Aspirin exposure was defined as the administration of an oral aspirin prescription within the first 3 days following admission to the ICU.

### 2.4 Data extraction

Demographic data such as age, gender, and ethnicity were collected. Vital signs measured upon admission included heart rate, mean blood pressure (MBP), and oxygen saturation (SpO_2_). Comorbidities recorded encompassed hypertension, diabetes, chronic pulmonary disease, and renal disease. Key laboratory indicators, such as white blood cell count (WBC), platelets, hemoglobin, glucose, blood urea nitrogen (BUN), potassium, and sodium levels were meticulously recorded. Disease severity was assessed using scores from the Glasgow Coma Scale (GCS), Sequential Organ Failure Assessment (SOFA), Charlson Comorbidity Index (CCI), and Systemic Inflammatory Response Syndrome (SIRS) score. Treatment interventions documented included mechanical ventilation (MV) and continuous renal replacement therapy (CRRT). Data on aspirin usage and other pertinent clinical interventions were also collected. Outcomes measured included 28-day and 90-day survival rates, in-hospital mortality, duration of ICU stay, and incidents of gastrointestinal hemorrhage and thrombocytopenia.

### 2.5 Primary outcome and secondary outcomes

This study’s primary outcome was 28-day mortality. Secondary outcomes included in-hospital mortality, 90-day mortality, duration of ICU stay, and the incidence of gastrointestinal hemorrhage and thrombocytopenia.

### 2.6 Statistical analysis

In our study, all variables exhibited less than 5% missing data, an imputation process was performed, employing the “mice” package in R software. Continuous variables are presented as mean (SD) for normally distributed data and as median (IQR) for non-normally distributed data. Categorical variables were expressed in numbers and percentages (%). The comparison of baseline data between the aspirin and non-aspirin groups was conducted using the t-test or Mann-Whitney U test for continuous variables and the Pearson Chi-square (χ2) test for categorical variables.

PSM was applied with a caliper width of 0.1 logits of the standard deviation to mitigate baseline imbalances. The cohort was paired in a 1:1 ratio using the nearest neighbor matching technique. The efficacy of the PSM was evaluated by the standardized mean difference (SMD), with an SMD ≤0.1 indicating a balanced model with regard to initial characteristics.

Survival curves for 28 days were generated using the Kaplan-Meier method to illustrate survival based on aspirin use and dosage, with differences assessed using the log-rank test. To assess the effect of aspirin on mortality, we established four models using multivariate Cox regression analysis, adjusting for different variables. Model 1 was unadjusted, serving as a baseline. Model 2 was adjusted for age, race, and renal disease. Model 3 included additional adjustments for heart rate, MBP, SpO2, hemoglobin, platelets, WBC, BUN, and potassium levels. Model 4 further adjusted for MV and CRRT. Results are presented as hazard ratios (HR) with 95% confidence intervals (CI). Furthermore, subgroups were classified by age, gender, race, and comorbidities, including chronic pulmonary disease, diabetes, hypertension, and renal disease, as well as interventions such as MV and CRRT. These subgroup analyses were intended to verify the consistency and robustness of our results. Interactions among subgroups were also investigated using a variance ratio test. All analyses were performed using R software version 4.3.2, with statistical significance set at *p* < 0.05.

## 3 Results

### 3.1 Patient characteristics

Our study enrolled 3,595 individuals diagnosed with CAP who met the inclusion criteria, including 2,261 treated with aspirin and 1,334 without aspirin treatment. Details regarding the dosage of aspirin used are presented in Supplementary Table S1. The most commonly used dosage was 85 mg/day (61.92%), followed by 325 mg/day (32.46%) and 300 mg/day (3.67%).

The clinical information of CAP patients in both groups, before and after PSM, is presented in Table 1. The average age and proportion of males in the aspirin treatment group were higher than those in the non-aspirin group. Patients receiving aspirin treatment showed a higher prevalence of chronic pulmonary disease (43.63%),hypertension (42.5%), diabetes (36.06%), and renal disease (33.21%). Additionally, the levels of WBC, platelets, glucose, BUN, and potassium were higher in the aspirin group, while hemoglobin and sodium levels showed no significant differences. In terms of disease severity scores, the aspirin group had a higher CCI (median [IQR]: 6 [4–8]) than the non-aspirin group (5 [3–7]) and a lower SIRS score (3 [2–3] vs. 3 [2–4]). There were no significant differences in GCS and SOFA between the groups.

**TABLE 1:** Baseline characteristics of CAP patients before and after PSM.

Variables	Before PSM					After PSM				
	Total (n = 3,595)	Non aspirin (n = 2,261)	Aspirin (n = 1,334)	*P*-value	SMD	Total (n = 2,438)	Non aspirin (n = 1,219)	Aspirin (n = 1,219)	*P*-value	SMD
Age	65.35 ± 16.56	62.14 ± 17.09	70.79 ± 14.06	<0.001	0.615	70.17 ± 14.26	70.18 ± 14.28	70.15 ± 14.25	0.957	−0.002
Gender, n (%)				0.047					0.775	
Female	1,589 (44.2)	1,028 (45.47)	561 (42.05)		−0.069	1,051 (43.11)	529 (43.40)	522 (42.82)		−0.012
Male	2,006 (55.8)	1,233 (54.53)	773 (57.95)		0.069	1,387 (56.89)	690 (56.60)	697 (57.18)		0.012
Race, n (%)				0.078					0.749	
Other	1,005 (27.96)	655 (28.97)	350 (26.24)		−0.062	653 (26.78)	323 (26.50)	330 (27.07)		0.013
White	2,590 (72.04)	1,606 (71.03)	984 (73.76)		0.062	1,785 (73.22)	896 (73.50)	889 (72.93)		−0.013
Chronic pulmonary disease, n (%)				<0.001					0.712	
No	2,204 (61.31)	1,452 (64.22)	752 (56.37)		−0.158	1,401 (57.47)	705 (57.83)	696 (57.10)		−0.015
Yes	1,391 (38.69)	809 (35.78)	582 (43.63)		0.158	1,037 (42.53)	514 (42.17)	523 (42.90)		0.015
Diabetes, n (%)				<0.001					0.223	
No	2,656 (73.88)	1,803 (79.74)	853 (63.94)		−0.329	1,666 (68.33)	847 (69.48)	819 (67.19)		−0.049
Yes	939 (26.12)	458 (20.26)	481 (36.06)		0.329	772 (31.67)	372 (30.52)	400 (32.81)		0.049
Hypertension, n (%)				<0.001					0.463	
No	2,231 (62.06)	1,464 (64.75)	767 (57.50)		−0.147	1,356 (55.62)	669 (54.88)	687 (56.36)		0.030
Yes	1,364 (37.94)	797 (35.25)	567 (42.50)		0.147	1,082 (44.38)	550 (45.12)	532 (43.64)		−0.030
Renal disease, n (%)				<0.001					0.475	
No	2,741 (76.24)	1,850 (81.82)	891 (66.79)		−0.319	1,732 (71.04)	874 (71.70)	858 (70.39)		−0.029
Yes	854 (23.76)	411 (18.18)	443 (33.21)		0.319	706 (28.96)	345 (28.30)	361 (29.61)		0.029
Heart rate (beats/min)	90.57 ± 16.50	91.98 ± 16.50	88.17 ± 16.24	<0.001	−0.234	88.84 ± 15.96	88.93 ± 15.69	88.75 ± 16.23	0.781	−0.011
MBP (mmHg)	76.33 ± 10.52	76.73 ± 10.73	75.66 ± 10.13	0.003	−0.106	75.99 ± 10.37	76.13 ± 10.54	75.86 ± 10.20	0.514	−0.027
SpO₂ (%)	96.45 ± 2.37	96.44 ± 2.49	96.48 ± 2.16	0.616	0.019	96.46 ± 2.32	96.46 ± 2.46	96.46 ± 2.17	0.993	0.000
Glucose (mg/dL)	130 (109, 161)	127 (107, 155)	138 (113, 171)	<0.001	0.226	135 (112, 166)	134 (112, 166)	136 (112, 167)	0.500	0.025
Hemoglobin (g/L)	9.9 (8.4, 11.4)	9.9 (8.4, 11.4)	9.9 (8.4, 11.5)	0.403	0.043	10.0 (8.5, 11.5)	10.0 (8.6, 11.5)	10.0 (8.4, 11.5)	0.782	0.002
Platelets (K/uL)	193 (126, 273)	185 (113, 267)	203 (141, 280)	<0.001	0.162	201 (137, 279.75)	201 (129, 283.50)	201 (140, 276)	0.610	0.015
WBC (K/uL)	9.6 (6.6, 13.5)	9.3 (6.3, 13.4)	9.9 (7.1, 13.6)	0.001	0.012	9.7 (6.9, 13.6)	9.6 (6.7, 13.7)	9.9 (7.0, 13.6)	0.338	−0.019
BUN (mg/dL)	21 (13, 35)	18 (12, 31)	25 (16, 41)	<0.001	0.299	24 (15, 38)	23 (14, 38)	24 (15, 38)	0.069	0.027
Sodium (mEq/L)	137 (134, 140)	137 (134, 140)	137 (134, 140)	0.669	−0.010	137 (134, 140)	137 (134, 140)	137 (134, 140)	0.498	−0.024
Potassium (mEq/L)	3.8 (3.5, 4.2)	3.8 (3.4, 4.2)	3.9 (3.5, 4.3)	<0.001	0.175	3.9 (3.5, 4.3)	3.9 (3.5, 4.3)	3.9 (3.5, 4.3)	0.259	−0.036
GCS	15 (13, 15)	15 (13, 15)	15 (13, 15)	0.136	−0.044	15 (13, 15)	15 (13, 15)	15 (13, 15)	0.739	−0.008
SOFA	5 (3, 8)	5 (3, 8)	5 (3, 8)	0.056	0.021	5 (3, 8)	5 (3, 8)	5 (3, 8)	0.535	−0.017
CCI	5 (3, 7)	5 (3, 7)	6 (4, 8)	<0.001	0.435	6 (4, 8)	5 (4, 8)	6 (4, 8)	0.225	0.042
SIRS score	3 (2, 4)	3 (2, 4)	3 (2, 3)	<0.001	−0.144	3 (2, 3)	3 (2, 3)	3 (2, 3)	0.832	−0.006
MV, n (%)				0.561					0.296	
No	2,488 (69.21)	1,557 (68.86)	931 (69.79)		0.020	1,668 (68.42)	822 (67.43)	846 (69.40)		0.043
Yes	1,107 (30.79)	704 (31.14)	403 (30.21)		−0.020	770 (31.58)	397 (32.57)	373 (30.60)		−0.043
CRRT, n (%)				0.137					0.880	
No	3,513 (97.72)	2,203 (97.43)	1,310 (98.20)		0.058	2,393 (98.15)	1,196 (98.11)	1,197 (98.20)		0.006
Yes	82 (2.28)	58 (2.57)	24 (1.80)		−0.058	45 (1.85)	23 (1.89)	22 (1.80)		−0.006

Abbreviations: CAP, community-acquired pneumonia; PSM, propensity score matching; SMD, standardized mean difference; MBP, mean blood pressure; SpO_2_, blood oxygen saturation; WBC, white blood cell count; BUN, blood urea nitrogen; GCS, glasgow coma scale; SOFA, sequential organ failure assessment; CCI, charlson comorbidity index; SIRS, systemic inflammatory response syndrome; MV, mechanical ventilation; CRRT, continuous renal replacement therapy.

**FIGURE 2:**
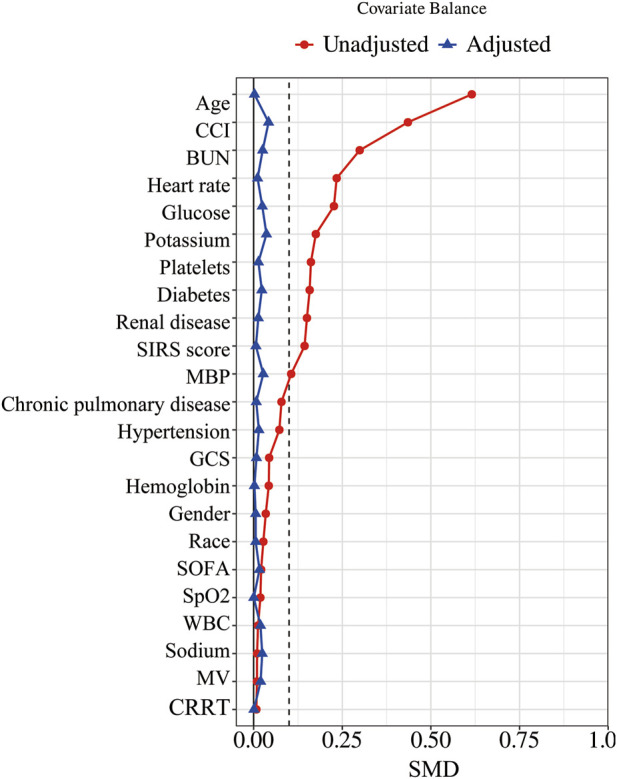
Standardized mean difference of variables before and after PSM. PSM, propensity score matching; SMD, standardized mean difference; MBP, mean blood pressure; SpO_2_, blood oxygen saturation; WBC, white blood cell count; BUN, blood urea nitrogen; GCS, Glasgow Coma Scale; SOFA, Sequential Organ Failure Assessment; CCI, Charlson Comorbidity Index; SIRS, Systemic Inflammatory Response Syndrome; MV, mechanical ventilation; CRRT, continuous renal replacement therapy.

In our study, the PSM method with a caliper value of 0.1 was applied to reduce the influence of confounding variables between the aspirin and non-aspirin groups. 1,219 pairs of CAP patients were matched. There were no statistical differences in the baseline characteristics between the two groups after PSM (Table 1; Figure 2).

### 3.2 Aspirin and primary outcomes

**FIGURE 3:**
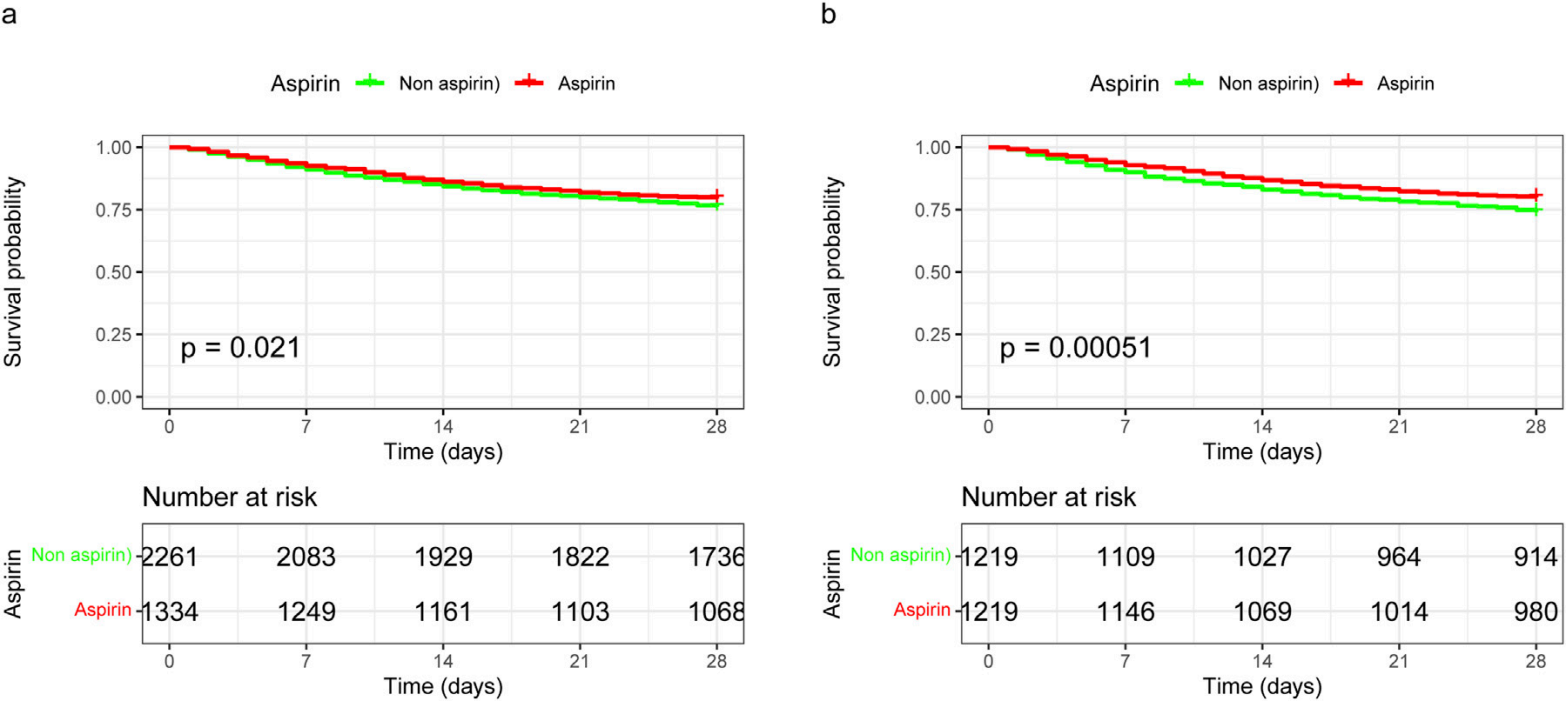
Kaplan-Meier survival curves for 28-day mortality following aspirin use in unmatched and propensity score-matched cohorts. **(A)** In the unmatched cohort, aspirin use was associated with improved 28-day survival, with a HR of 0.84 (95% CI 0.73–0.98). **(B)** In the propensity score-matched cohort, aspirin use was associated with further improved 28-day survival, with a HR of 0.75 (95% CI 0.63–0.88). HR, hazard ratio; 95% CI, 95% confidence interval.

The 28-day mortality rate for aspirin users was 20.46%, which was lower than that observed in non-users (Table 2). Kaplan–Meier survival analysis revealed a significant improvement in 28-day survival in the aspirin group in both the unmatched cohort and the propensity score-matched cohort (Figure 3). Supplementary Table S2 compares the baseline characteristics of patients with 28-day survival and those with 28-day mortality. Univariate Cox regression analysis was performed to identify risk factors for 28-day mortality (Supplementary Table S3). We developed four multivariate Cox regression models to assess the independent impact of aspirin treatment on 28-day mortality. The HRs and 95% CIs are detailed in Table 3. Aspirin treatment was significantly associated with decreased 28-day mortality in both the pre-PSM (HR 0.71, 95% CI 0.61–0.82, *p* < 0.001) and post-PSM cohorts (HR 0.75, 95% CI 0.63–0.88, *p* < 0.001).

**TABLE 2:** Association between aspirin and clinical outcomes in CAP.

Variables	Total	Non aspirin	Aspirin	*P*-value	HR/OR (95% CI)
Before PSM	n = 3,595	n = 2,261	n = 1,334		
Primary outcome					
28-day mortality, n (%)	812 (22.59)	539 (23.84)	273 (20.46)	0.019	0.84 (0.73–0.98)
Secondary outcomes					
Hospital mortality, n (%)	738 (20.53)	479 (21.19)	259 (19.42)	0.204	0.90 (0.76–1.06)
90-day mortality, n (%)	1,188 (33.05)	747 (33.04)	441 (33.06)	0.990	0.98 (0.87–1.10)
ICU stay (days)	3.77 (1.98, 8.30)	3.71 (1.97, 8.19)	3.94 (2.01, 8.47)	0.263	
Gastrointestinal hemorrhage, n (%)	96 (2.67)	59 (2.61)	37 (2.77)	0.768	1.06 (0.70–1.62)
Thrombocytopenia, n (%)	568 (15.80)	374 (16.54)	194 (14.54)	0.112	0.86 (0.71–1.04)
After PSM	n = 2,438	n = 1,219	n = 1,219		
Primary outcome					
28-day mortality, n (%)	562 (23.05)	317 (26.00)	245 (20.10)	<0.001	0.75 (0.63–0.88)
Secondary outcomes					
Hospital mortality, n (%)	509 (20.88)	274 (22.48)	235 (19.28)	0.052	0.82 (0.68–1.00)
90-day mortality, n (%)	846 (34.70)	444 (36.42)	402 (32.98)	0.074	0.87 (0.76–0.99)
ICU stay (days)	3.82 (1.98, 8.24)	3.66 (1.94, 8.01)	4.01 (2.03, 8.63)	0.090	
Gastrointestinal hemorrhage, n (%)	65 (2.67)	30 (2.46)	35 (2.87)	0.530	1.17 (0.71–1.92)
Thrombocytopenia, n (%)	350 (14.36)	169 (13.86)	181 (14.85)	0.488	1.08 (0.86–1.36)

Abbreviations: CAP, community-acquired pneumonia; PSM, propensity score matching; HR, hazard ratio; OR, odds ratio; 95% CI, 95% confidence interval; ICU, intensive care unit.

**TABLE 3:** Association of aspirin and the risk of 28-day mortality.

Models	Before PSM			After PSM		
	HR	95% CI	*P*-value	HR	95% CI	*P*-value
Model 1	0.84	0.73–0.98	0.022	0.75	0.63–0.88	<0.001
Model 2	0.69	0.59–0.80	<0.001	0.75	0.63–0.88	<0.001
Model 3	0.71	0.61–0.82	<0.001	0.74	0.63–0.88	<0.001
Model 4	0.71	0.61–0.82	<0.001	0.75	0.63–0.88	<0.001

Model 1: unadjusted.

Model 2: adjusted for age, race, renal disease.

Model 3: adjusted for age, race, renal disease, heart rate, MBP, SpO_2_, hemoglobin, platelets, WBC, BUN, potassium.

Model 4: adjusted for age, race, renal disease, heart rate, MBP, SpO_2_, hemoglobin, platelets, WBC, BUN, potassium, MV, CRRT.

Abbreviations: PSM, propensity score matching; HR, hazard ratio; 95% CI, 95% confidence interval; MBP, mean blood pressure; SpO_2_, blood oxygen saturation; WBC, white blood cell count; BUN, blood urea nitrogen; MV, mechanical ventilation; CRRT, continuous renal replacement therapy.

### 3.3 Aspirin and secondary outcomes

There were no significant differences between the aspirin and non-aspirin groups in terms of hospital mortality, 90-day mortality, ICU stay, gastrointestinal hemorrhage, and thrombocytopenia before PSM. After PSM, the aspirin group exhibited slightly lower hospital mortality compared to the non-aspirin group (19.28% vs 22.48%, *p* = 0.052) and lower 90-day mortality (32.98% vs 36.42%, *p* = 0.074). However, no significant differences were observed in ICU stay, gastrointestinal hemorrhage, and thrombocytopenia between the two groups (Table 2).

### 3.4 Dose of aspirin and outcomes

Among patients using aspirin, 81 mg/day and 325 mg/day were the most common dosages. Therefore, we chose to analyze the impact of these two dosages on patient outcomes. We applied the same principles and methods for PSM as used for comparing aspirin and non-aspirin users to the different dosage groups. The results are presented in Supplementary Table S4 and Supplementary Figure S1.

**FIGURE 4:**
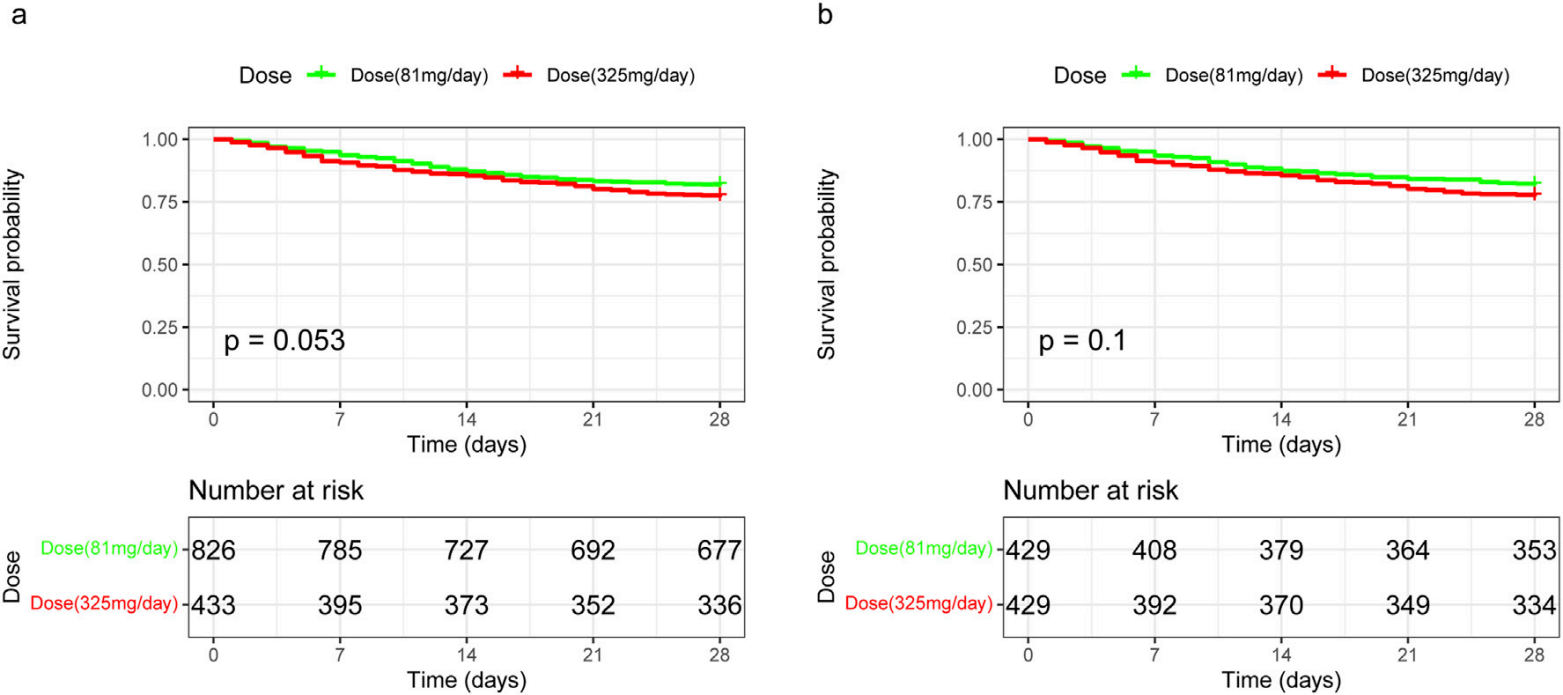
Kaplan-Meier survival curves for 28-day mortality following varying doses of aspirin in unmatched and propensity score-matched cohorts. **(A)** In the unmatched cohort, 325 mg/day of aspirin demonstrated a trend towards worsened 28-day survival, although this was not statistically significant, with a HR of 1.28 (95% CI 1.00–1.65). **(B)** In the propensity score-matched cohort, the difference in 28-day survival between the 325 mg/day and 81 mg/day aspirin dosages remained statistically non-significant, with an HR of 1.28 (95% CI 0.95–1.72). HR, hazard ratio; 95% CI, 95% confidence interval.

Kaplan-Meier survival curves for 28-day mortality following varying doses of aspirin showed that 325 mg/day demonstrated a trend towards improved 28-day survival before matching (*p* = 0.053), but no significant difference after matching (*p* = 0.1) compared to 81 mg/day (Figure 4). Additionally, there were no differences between 325 mg/day and 81 mg/day aspirin treatments in terms of 28-day mortality, hospital mortality, 90-day mortality, gastrointestinal hemorrhage, and thrombocytopenia. However, ICU stay was longer for the 325 mg/day group compared to the 81 mg/day group (4.22 vs. 3.57 days, *p* = 0.031) after PSM (Table 4).

**TABLE 4:** Association between aspirin dose and clinical outcomes in CAP.

Variables	Total	Dose (81 mg/day)	Dose (325 mg/day)	*P*-value	HR/OR (95% CI)
Before PSM	n = 1,259	n = 826	n = 433		
Primary outcome					
28-day mortality, n (%)	253 (20.10)	153 (18.52)	100 (23.09)	0.054	1.28 (1.00–1.65)
Secondary outcomes					
Hospital mortality, n (%)	241 (19.14)	150 (18.16)	91 (21.02)	0.221	1.20 (0.90–1.60)
90-day mortality, n (%)	414 (32.88)	257 (31.11)	157 (36.26)	0.065	1.21 (0.99–1.48)
ICU stay (days)	3.91 (2.00, 8.18)	3.75 (2.01, 7.86)	4.26 (1.94, 9.06)	0.102	
Gastrointestinal hemorrhage, n (%)	35 (2.78)	22 (2.66)	13 (3.00)	0.728	1.13 (0.56–2.27)
Thrombocytopenia, n (%)	188 (14.93)	123 (14.89)	65 (15.01)	0.955	1.01 (0.73–1.40)
After PSM	n = 858	n = 429	n = 429		
Primary outcome					
28-day mortality, n (%)	177 (20.63)	79 (18.41)	98 (22.84)	0.109	1.28 (0.95–1.72)
Secondary outcomes					
Hospital mortality, n (%)	170 (19.81)	81 (18.88)	89 (20.75)	0.493	1.12 (0.80–1.57)
90-day mortality, n (%)	293 (34.15)	138 (32.17)	155 (36.13)	0.221	1.16 (0.92–1.46)
ICU stay (days)	3.87 (1.94, 8.11)	3.57 (1.95, 7.69)	4.22 (1.94, 9.06)	0.031	
Gastrointestinal hemorrhage, n (%)	21 (2.45)	8 (1.86)	13 (3.03)	0.269	1.64 (0.6–4.01)
Thrombocytopenia, n (%)	124 (14.45)	59 (13.75)	65 (15.15)	0.560	1.12 (0.77–1.64)

Abbreviations: CAP, community-acquired pneumonia; PSM, propensity score matching; HR, hazard ratio; OR, odds ratio; 95% CI, 95% confidence interval; ICU, intensive care unit.

### 3.5 Subgroup analysis

**FIGURE 5:**
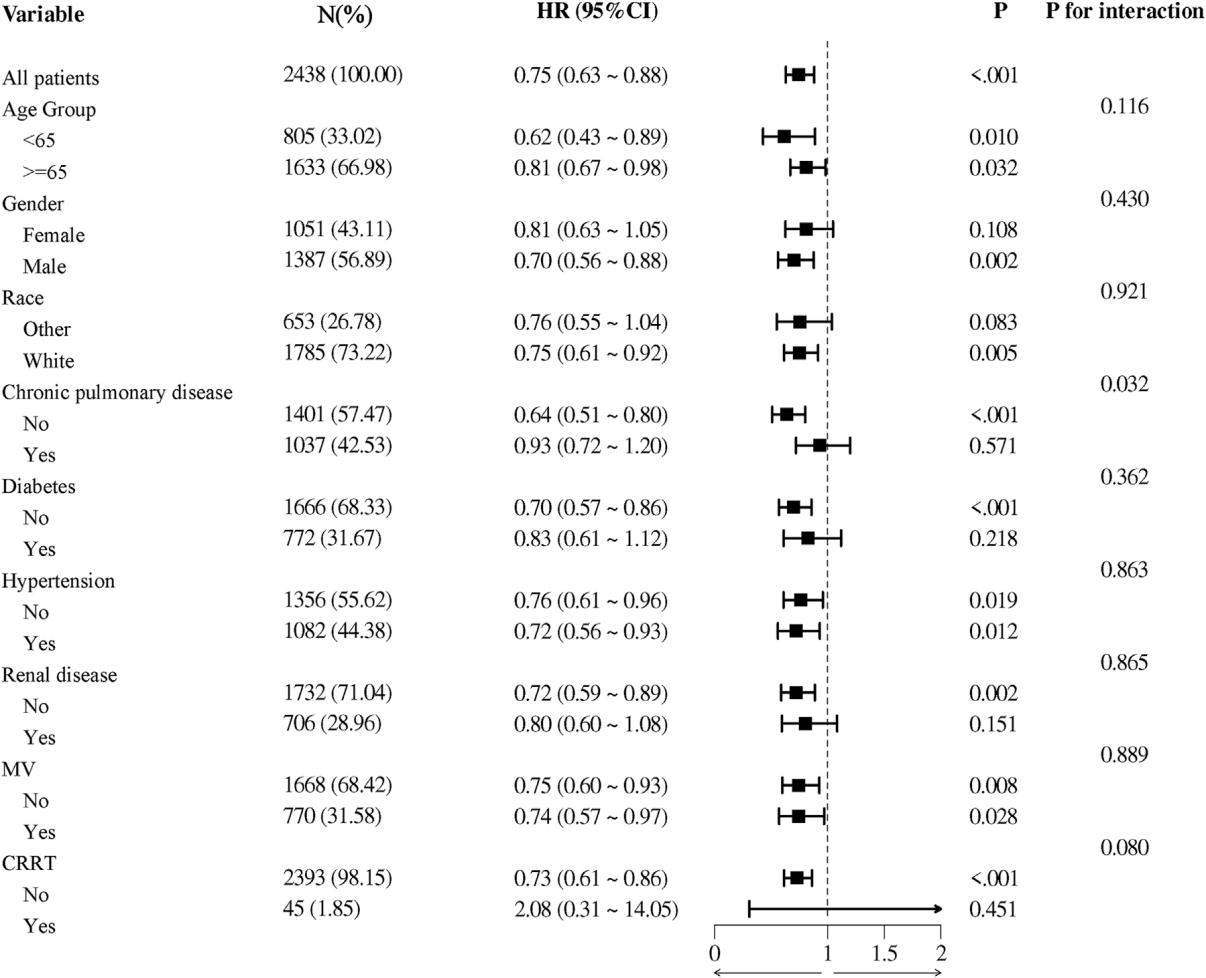
The association between aspirin treatment and 28-day mortality in subgroups. HRs adjusted for age, race, renal disease, heart rate, MBP, SpO_2_, hemoglobin, platelets, WBC, BUN, potassium, MV, CRRT. HR, hazard ratio; 95% CI, 95% confidence interval; MBP, mean blood pressure; SpO_2_, blood oxygen saturation; WBC, white blood cell count; BUN, blood urea nitrogen; MV, mechanical ventilation; CRRT, continuous renal replacement therapy.

In the subgroup analysis, we found that aspirin had a significant interaction effect in subgroup of chronic pulmonary disease (*p* for interaction = 0.032). Aspirin use showed no significant interaction effect in age, gender, race, diabetes, hypertension, renal disease, MV and CRRT subgroups (Figure 5).

## 4 Discussion

Current studies on aspirin use in patients with CAP suggest potential benefits, particularly in reducing cardiovascular complications and mortality. A notable study demonstrated that combining aspirin with macrolides in patients with severe CAP significantly improved 30-day survival rates, evidenced by a HR of 0.71 (95% CI 0.58–0.88, *p* = 0.002) (Falcone et al., 2019). Further research utilizing extensive primary care databases revealed that aspirin markedly reduced the risk of combined cardiovascular outcomes (HR 0.70, 95% CI 0.55–0.91, *p* < 0.05) following CAP diagnosis (Hamilton et al., 2021). Additionally, aspirin use in hospitalized CAP patients was associated with a significantly reduced 30-day mortality (HR 0.43, 95% CI 0.25–0.75, *p* = 0.003) (Falcone et al., 2015), and in individuals with pre-existing cardio-cerebral vascular diseases, long-term low-dose aspirin use modestly decreased the incidence of pneumonia (HR 0.89, 95% CI 0.84–0.95, *p* < 0.001) (Chen et al., 2021). Our observational study conducted using the MIMIC IV database further substantiates the benefits of aspirin in CAP management. We found that aspirin treatment was associated with improved 28-day survival in a propensity score-weighted model, presenting an HR of 0.75 (95% CI 0.63–0.88, *p* < 0.001). Crucially, this improvement in survival was achieved without an increase in the risks of gastrointestinal hemorrhage or thrombocytopenia. Moreover, our findings elucidate an important aspect of aspirin dosing: while a daily dose of 325 mg did not provide additional benefits, it correlated with a longer ICU stay compared to a daily dose of 81 mg (4.22 vs 3.57 days, *p* = 0.031). This dosing distinction underscores the clinical implications of aspirin usage in CAP, suggesting that lower doses may optimize patient outcomes and enhance safety.

Aspirin, a classic antiplatelet agent, is widely used in clinical practice. Extensive clinical trials have consistently shown aspirin’s efficacy in reducing the risk of myocardial infarction, stroke, and other cardiovascular events (Gaziano et al., 2018; Zheng and Roddick, 2019). By irreversibly inhibiting COX-1 and reducing thromboxane A2 (TXA2) production, it effectively prevents platelet aggregation and thrombus formation (Fontana et al., 2014). About 30% of CAP patients face a risk of cardiovascular events such as heart failure, atrial fibrillation, and myocardial infarction (Corrales-Medina et al., 2012). Aspirin can improve the prognosis of CAP patients by reducing cardiovascular events through anti-platelet activation. A multi-center, prospective, randomized trial with 185 CAP patients at cardiovascular risk showed prophylactic aspirin treatment significantly reduced acute coronary syndrome risk and mortality (Oz et al., 2013). Additionally, Hamilton performed a PSM analysis in a large primary care database, revealing a significant reduction in cardiovascular events among aspirin user 6 months after pneumonia (493 vs. 877 events), and that previous aspirin use is significantly associated with a decrease in myocardial infarction and stroke (HR 0.64, 95% CI 0.52–0.79) (Hamilton et al., 2021).

Beyond its antiplatelet effects, researchers have discovered aspirin possesses additional mechanisms that improve the survival of patients with CAP. Aspirin has been shown to reduce the release of specific inflammatory mediators, such as lipoxin A4 (LXA4), synthesis of pro-resolving lipid mediators (SPM), interleukin-1 beta (IL-1β), and interleukin-6 (IL-6), and to promote their decomposition (Rood et al., 2023). Wang et al. showed that aspirin can reduce the load of pneumococcal bacteria, promote the faster clearance of pneumococcal bacteria in the lungs, and limit the excessive leukocyte chemotaxis from the infected bronchioles to the distal regions of the lungs by triggering the release of aspirin-triggered resolvin D1 (Wang et al., 2017). In addition, aspirin might enhance arterial oxygenation by modifying pulmonary blood flow’s regional distribution. In animal models with lobar pneumonia induced by pneumococcal infection, researchers noted a decrease in intrapulmonary shunt flow and an increase in oxygen’s partial pressure 60 min post aspirin’s intravenous administration (Ferrer et al., 1997). Moreover, aspirin may have direct antibacterial effects and modulate bacterial virulence. Lee et al. showed that aspirin reduces *Klebsiella pneumoniae*’s resistance to neutrophil phagocytosis and boosts the bactericidal activity of leukocytes (Lee et al., 2014).

One interesting finding in our study was the significantly higher prevalence of chronic respiratory diseases among ICU patients with CAP who were prescribed aspirin compared to those who were not (43.63% vs 35.78%, *p* < 0.001). This association can be attributed to several factors. Patients with chronic respiratory conditions, often have elevated cardiovascular risk, which leads to more frequent use of aspirin to prevent thrombotic events (Yang et al., 2024). Additionally, ICU patients with chronic respiratory diseases typically receive more intensive medical care, increasing the likelihood of aspirin being prescribed to address both their respiratory and cardiovascular needs (Shen et al., 2021). Another notable finding was that levels of WBC, platelets, glucose, BUN, and potassium were higher in the aspirin group. This finding may be attributed to the baseline health conditions of these patients. Our study found that the aspirin group had a higher prevalence of chronic pulmonary disease, hypertension, diabetes, and renal disease. These chronic conditions are well-known to be associated with elevated levels of these biomarkers. For example, diabetes is often linked with higher glucose levels due to insulin resistance and metabolic dysregulation (Samuel and Shulman, 2016). Chronic pulmonary disease can increase inflammatory markers, resulting in elevated WBC and platelet counts (El-Gazzar et al., 2020). Additionally, renal disease is frequently associated with increased BUN and potassium levels due to impaired kidney function (Stevens et al., 2013). These underlying health conditions likely account for the observed higher baseline levels of these biomarkers in the aspirin group.

In clinical practice, aspirin is predominantly prescribed at two standard dosages: 81 mg/day, commonly known as low-dose aspirin, and 325 mg/day, regarded as the standard therapeutic dose (Qayyum et al., 2008). The primary use of aspirin in CAP patients is focused on its antiplatelet properties, which reduce cardiovascular events, and its anti-inflammatory effects (Falcone et al., 2015; Hybiak et al., 2020). In this study, we observed that 81 mg/day and 325 mg/day were the most commonly prescribed doses of aspirin among patients treated for CAP, aligning with established clinical practices. Unfortunately, due to the nature of the MIMIC database, we were unable to determine the reasons behind the physicians’ choice of different dosages. After using PSM to adjust for baseline differences, we observed no significant differences between the 325 mg/day and 81 mg/day groups in terms of 28-day mortality, hospital mortality, 90-day mortality, gastrointestinal hemorrhage, and thrombocytopenia. However, ICU stay was longer for the 325 mg/day group compared to the 81 mg/day group (4.22 vs. 3.57 days, *p* = 0.031). The effect of aspirin is dose-dependent (Vane, 1971). At low dose, aspirin irreversibly inhibits COX-1, which is crucial in reducing platelet aggregation and the secretion of TXA2, thereby providing significant antiplatelet effects (Fontana et al., 2014). These effects can reduce the risk of thrombotic events, which are a concern in patients with CAP due to increased inflammation and coagulation during infection. At higher dose, aspirin inhibits both COX-1 and COX-2, enhancing its utility by offering both antiplatelet and anti-inflammatory effects (Christiansen et al., 2021). This broader inhibition is beneficial in reducing the inflammatory responses that are prevalent in CAP, potentially leading to improved outcomes by mitigating pulmonary inflammation and associated systemic effects. Our findings suggest that administering 325 mg/day of aspirin did not offer additional benefits over a lower dose of 81 mg/day in patients with CAP. We speculate that the observed improvements in CAP patient outcomes are primarily due to aspirin’s antiplatelet effects. These are crucial, as they help reduce the risk of cardiovascular events, a significant concern in CAP due to heightened inflammation and the potential for increased coagulation during infection. Although our research indicated that higher dose aspirin do not increase the risk of gastrointestinal hemorrhage and thrombocytopenia, it did increase the ICU stay time. Therefore, the use of 81 mg/day aspirin may be more appropriate.

Our study observed that the 325 mg/day aspirin treatment group had longer ICU stays. Although our population differs, supporting evidence from other studies suggests that high-dose aspirin may contribute to cardiovascular complications, which could explain the prolonged hospitalization. For example, an observational study involving 30,827 individuals already at risk for heart failure reported an increased risk of developing heart failure associated with high-dose aspirin use (HR 1.26, 95% CI 1.12–1.41, *p* < 0.001) (Mujaj et al., 2022). Additionally, a meta-analysis of 25,083 patients who underwent Percutaneous Coronary Intervention (PCI) found that higher aspirin doses (≥200 mg/day) were associated with a significantly increased incidence of major adverse cardiac events (OR 1.20, 95% CI 1.02–1.41, *p* = 0.03) (Bundhun et al., 2016). These studies, although not specific to CAP populations, indicate that higher doses of aspirin can elevate the risk of cardiovascular events, which may in turn lead to longer ICU stays. Moreover, the use of a higher dose of aspirin results in an extended period for coagulation parameters to normalize (Boss et al., 1985). Since these parameters are crucial indicators of a patient’s readiness for discharge, their delayed normalization consequently extends the time required for patients to meet discharge criteria. Subgroup analysis showed an interaction effect between aspirin treatment and 28-day prognosis in the chronic pulmonary disease group (*p* for interaction = 0.032), and patients without chronic pulmonary disease had a lower HR, suggesting that aspirin was more effective in patients without chronic pulmonary disease. This difference may be due to the altered pharmacokinetics in patients with chronic pulmonary diseases, which can impact hepatic blood flow and consequently the metabolism of drugs like aspirin (Taburet et al., 1990). Additionally, chronic inflammation, a common characteristic of pulmonary diseases, can alter platelet function and contribute to aspirin resistance (Mallah et al., 2020). This resistance is defined as the failure of aspirin to achieve the expected inhibition of platelet aggregation (Hankey and Eikelboom, 2006). Pathophysiologically, the persistent inflammatory state associated with chronic pulmonary disease can enhance the production of TXA2, a potent platelet activator, thereby reducing the efficacy of aspirin’s antiplatelet action (Floyd and Ferro, 2014). Consequently, this resistance might diminish the effectiveness of aspirin, making it less beneficial for patients with chronic lung diseases compared to those without such conditions.

It should be noted that in the ICU setting, patients routinely receive medications related to their chronic conditions, which might interact with aspirin, impacting both its therapeutic effect and safety profile. For instance, patients with diabetes may be treated with insulin or other hypoglycemic agents, which could alter inflammatory responses and coagulation statuses, thereby indirectly modifying the action of aspirin (Kothari et al., 2016). Similarly, individuals with chronic pulmonary diseases, such as chronic obstructive pulmonary disease (COPD) and asthma, might be on corticosteroids like prednisone or budesonide. These corticosteroids can interact with aspirin’s anti-inflammatory and anticoagulant properties, leading to an increased risk of gastrointestinal ulcers and bleeding due to the combined suppression of prostaglandin synthesis, which protects the gastric mucosa (Saxena et al., 2013). To delve deeper into the potential impacts of polypharmacy on the efficacy of aspirin in treating CAP, we employed PSM to minimize baseline discrepancies between the aspirin-treated and untreated groups. Although our results indicate an association between aspirin usage and decreased mortality, future research should explore the specific impacts of various drug combinations on the prognosis of CAP patients.

However, this study has some limitations. Firstly, the potential for selection bias cannot be overlooked. Firstly, our research relies on the MIMIC database, which comprises patients from specific intensive care units. This sample may not adequately represent the broader population of CAP patients, potentially limiting the generalizability of our findings regarding the efficacy of aspirin in reducing 28-day mortality. Expanding future research to include additional medical databases, such as the Healthcare Cost and Utilization Project (HCUP) and the Veterans Health Administration (VHA), could help to validate our observations across a more diverse cohort and provide a more comprehensive understanding of aspirin’s impact on CAP outcomes. Secondly, while PSM aims to adjust for observable confounders, the presence of unmeasured confounding variables remains a critical challenge. These variables, not captured in the database, could affect the study’s ability to establish a definitive causal relationship between aspirin use and reduced mortality. Thirdly, some patients may have been admitted to the ICU for other acute conditions that also necessitated intensive care, but were concurrently affected by CAP during their hospital stay. These conditions include but are not limited to acute myocardial infarction, congestive heart failure, acute kidney injury, and sepsis originating from sources other than the lungs (Dai et al., 2020). This co-morbidity introduces additional complexity in interpreting the direct impact of aspirin on CAP outcomes. Lastly, essential inflammation markers such as C-reactive protein (CRP), Procalcitonin (PCT), and IL-6 were not included in our analysis due to significant data unavailability. These markers are critical for comprehensively understanding the inflammatory status and clinical severity of CAP (Karhu et al., 2019).

In conclusion, our study demonstrates that aspirin reduces the 28-day mortality rates among CAP patients and does not increase the risk of gastrointestinal hemorrhage or thrombocytopenia. Moreover, 325 mg/day aspirin does not provide extra benefits over 81 mg/day and may lead to longer ICU stays. Although aspirin shows potential as a treatment for CAP, randomized clinical trials are needed to support this finding.

## Data Availability

The raw data supporting the conclusions of this article will be made available by the authors, without undue reservation.
