# Supplementary material for: Aspirin reduces the mortality risk of patients with community-acquired pneumonia: a retrospective propensity-matched analysis of the MIMIC-IV database
Source: Front Pharmacol. 2024 Sep 13;15:1402386. doi: 10.3389/fphar.2024.1402386 (PMC11427301; doi:10.3389/fphar.2024.1402386)
Supplement: Supplementary file 1 [file Table1.DOCX]

Table S1. Distribution of aspirin dosage for treatment.

| Aspirin dose(mg/day) | n(%) |
| --- | --- |
| 80 | 10(0.75) |
| 81 | 826(61.92) |
| 100 | 1(0.075) |
| 150 | 1(0.075) |
| 162 | 10(0.75) |
| 300 | 49(3.67) |
| 324 | 1(0.075) |
| 325 | 433(32.46) |
| 600 | 2(0.15) |
| 650 | 1(0.075) |

Table S2. Baseline characteristics of patients at 28-day survival and 28-day mortality.

| **Variables** | **Total**  **(n = 3,595)** | **28-day survival**  **(n = 2,783)** | **28-day mortality**  **(n = 812)** | **P value** |
| --- | --- | --- | --- | --- |
|  |  |  |  |  |
| Age | 65.35 ± 16.56 | 63.87 ± 16.69 | 70.43 ± 15.06 | <0.001 |
| Gender, n(%) |  |  |  | 0.694 |
| Female | 1,589 (44.20) | 1,235 (44.38) | 354 (43.60) |  |
| Male | 2,006 (55.80) | 1,548 (55.62) | 458 (56.40) |  |
| Race, n(%) |  |  |  | 0.021 |
| Other | 1,005 (27.96) | 752 (27.02) | 253 (31.16) |  |
| White | 2,590 (72.04) | 2,031 (72.98) | 559 (68.84) |  |
| Chronic pulmonary disease, n(%) |  |  |  | 0.258 |
| No | 2,204 (61.31) | 1,720 (61.80) | 484 (59.61) |  |
| Yes | 1,391 (38.69) | 1,063 (38.20) | 328 (40.39) |  |
| Diabetes, n(%) |  |  |  | 0.723 |
| No | 2,656 (73.88) | 2,060 (74.02) | 596 (73.40) |  |
| Yes | 939 (26.12) | 723 (25.98) | 216 (26.60) |  |
| Hypertension, n(%) |  |  |  | 0.327 |
| No | 2,231 (62.06) | 1,739 (62.49) | 492 (60.59) |  |
| Yes | 1,364 (37.94) | 1,044 (37.51) | 320 (39.41) |  |
| Renal disease, n(%) |  |  |  | 0.011 |
| No | 2,741 (76.24) | 2,149 (77.22) | 592 (72.91) |  |
| Yes | 854 (23.76) | 634 (22.78) | 220 (27.09) |  |
| Heart rate (beats/min) | 90.57 ± 16.50 | 89.74 ± 16.13 | 93.42 ± 17.43 | <0.001 |
| MBP(mmHg) | 76.33 ± 10.52 | 77.05 ± 10.41 | 73.89 ± 10.55 | <0.001 |
| SpO_2_ (%) | 96.45 ± 2.37 | 96.56 ± 2.19 | 96.07 ± 2.90 | <0.001 |
| Glucose (mg/dL) | 130 (109, 161) | 130 (109, 160) | 133 (109, 162) | 0.447 |
| Hemoglobin (g/L) | 9.9 (8.4, 11.4) | 10.0 (8.5, 11.5) | 9.45 (8.1, 11.0) | <0.001 |
| Platelets (K/uL) | 193 (126, 273) | 196 (131, 273) | 179 (107, 271) | 0.001 |
| WBC (K/uL) | 9.6 (6.6, 13.5) | 9.4 (6.6, 12.9) | 10.5 (6.6, 15.6) | <0.001 |
| BUN (mg/dL) | 21 (13, 35) | 19 (12, 32) | 28 (17, 45) | <0.001 |
| Sodium (mEq/L) | 137 (134, 140) | 137 (134, 140) | 136 (133, 140) | 0.074 |
| Potassium (mEq/L) | 3.8 (3.5, 4.2) | 3.8 (3.5, 4.2) | 3.9 (3.5, 4.4) | 0.003 |
| GCS | 15 (13, 15) | 15 (14, 15) | 14.50 (12, 15) | <0.001 |
| SOFA | 5 (3, 8) | 5 (3, 7) | 7 (4, 10) | <0.001 |
| CCI | 5 (3, 7) | 5 (3, 7) | 6 (5, 8) | <0.001 |
| SIRS score | 3 (2, 4) | 3 (2, 4) | 3 (3, 4) | <0.001 |
| MV, n(%) |  |  |  | <0.001 |
| No | 2,488 (69.21) | 2,006 (72.08) | 482 (59.36) |  |
| Yes | 1,107 (30.79) | 777 (27.92) | 330 (40.64) |  |
| CRRT, n(%) |  |  |  | <0.001 |
| No | 3,513 (97.72) | 2,732 (98.17) | 781 (96.18) |  |
| Yes | 82 (2.28) | 51 (1.83) | 31 (3.82) |  |
| Aspirin treatment, n(%) |  |  |  |  |
| No | 2,261 (62.89) | 1,722 (61.88) | 539 (66.38) | 0.019 |
| Yes | 1,334 (37.11) | 1,061 (38.12) | 273 (33.62) |  |

Abbreviations: MBP, mean blood pressure; SpO_2_, blood oxygen saturation; WBC, white blood cell count; BUN, blood urea nitrogen; GCS, Glasgow Coma Scale; SOFA, Sequential Organ Failure Assessment; CCI, Charlson Comorbidity Index; SIRS, Systemic Inflammatory Response Syndrome; MV, mechanical ventilation; CRRT, continuous renal replacement therapy.

Table S3. Univariate cox regression analysis of factors influencing 28-day mortality.

| **Variables** | **HR (95% CI)** | **P value** |
| --- | --- | --- |
| Age | 1.02 (1.02-1.03) | <.001 |
| Gender |  |  |
| Female | 1.00 (Reference) |  |
| Male | 1.02 (0.89-1.18) | 0.740 |
| Race |  |  |
| Other | 1.00 (Reference) |  |
| White | 0.83 (0.72-0.97) | 0.016 |
| Renal Disease |  |  |
| No | 1.00 (Reference) |  |
| Yes | 1.21 (1.04-1.42) | 0.014 |
| Heart Rate | 1.01 (1.01-1.02) | <.001 |
| MBP | 0.97 (0.96-0.98) | <.001 |
| SpO_2_ | 0.92 (0.90-0.95) | <.001 |
| Hemoglobin | 0.94 (0.91-0.97) | <.001 |
| Platelets | 0.99 (0.99-0.99) | 0.021 |
| WBC | 1.01 (1.01-1.02) | <.001 |
| BUN | 1.01 (1.01-1.02) | <.001 |
| Potassium | 1.22 (1.09-1.37) | <.001 |
| MV |  |  |
| No | 1.00 (Reference) |  |
| Yes | 1.68 (1.46-1.93) | <.001 |
| CRRT |  |  |
| No | 1.00 (Reference) |  |
| Yes | 1.87 (1.31-2.68) | <.001 |
| Aspirin treatment |  |  |
| No | 1.00 (Reference) |  |
| Yes | 0.84(0.73-0.98) | 0.022 |

Abbreviations: HR, hazard ratio; 95% CI, 95% confidence interval; MBP, mean blood pressure; SpO_2_, blood oxygen saturation; WBC, white blood cell count; BUN, blood urea nitrogen; MV, mechanical ventilation; CRRT, continuous renal replacement therapy.

Table S4. Baseline characteristics of patients receiving 81 mg/day and 325 mg/day aspirin before and after PSM.

| **Variables** | **Before PSM** | | | | | **After PSM** | | | | |
| --- | --- | --- | --- | --- | --- | --- | --- | --- | --- | --- |
|  | **Total**  **(n = 1,259)** | **Dose(81mg/day)**  **(n = 826)** | **Dose(325mg/day)**  **(n = 433)** | **P value** | **SMD** | **Total**  **(n = 858)** | **Dose(81mg/day)**  **(n = 429)** | **Dose(325mg/day)**  **(n = 429)** | **P value** | **SMD** |
| Age | 70.93 ± 13.92 | 70.93 ± 13.80 | 70.91 ± 14.18 | 0.983 | -0.001 | 71.38 ± 13.89 | 71.84 ± 13.69 | 70.91 ± 14.09 | 0.328 | -0.066 |
| Gender, n (%) |  |  |  | 0.262 |  |  |  |  | 1.000 |  |
| Female | 534 (42.41) | 341 (41.28) | 193 (44.57) |  | 0.066 | 382 (44.52) | 191 (44.52) | 191 (44.52) |  | 0.000 |
| Male | 725 (57.59) | 485 (58.72) | 240 (55.43) |  | -0.066 | 476 (55.48) | 238 (55.48) | 238 (55.48) |  | 0.000 |
| Race, n (%) |  |  |  | 0.132 |  |  |  |  | 0.936 |  |
| Other | 332 (26.37) | 229 (27.72) | 103 (23.79) |  | -0.092 | 205 (23.89) | 103 (24.01) | 102 (23.78) |  | -0.005 |
| White | 927 (73.63) | 597 (72.28) | 330 (76.21) |  | 0.092 | 653 (76.11) | 326 (75.99) | 327 (76.22) |  | 0.005 |
| Chronic pulmonary disease, n (%) |  |  |  | 0.193 |  |  |  |  | 0.409 |  |
| No | 701 (55.68) | 449 (54.36) | 252 (58.20) |  | 0.078 | 484 (56.41) | 236 (55.01) | 248 (57.81) |  | 0.057 |
| Yes | 558 (44.32) | 377 (45.64) | 181 (41.80) |  | -0.078 | 374 (43.59) | 193 (44.99) | 181 (42.19) |  | -0.057 |
| Diabetes, n (%) |  |  |  | 0.088 |  |  |  |  | 0.772 |  |
| No | 806 (64.02) | 515 (62.35) | 291 (67.21) |  | 0.103 | 570 (66.43) | 283 (65.97) | 287 (66.90) |  | 0.020 |
| Yes | 453 (35.98) | 311 (37.65) | 142 (32.79) |  | -0.103 | 288 (33.57) | 146 (34.03) | 142 (33.10) |  | -0.020 |
| Hypertension, n (%) |  |  |  | 0.524 |  |  |  |  | 0.837 |  |
| No | 722 (57.35) | 479 (57.99) | 243 (56.12) |  | -0.038 | 479 (55.83) | 238 (55.48) | 241 (56.18) |  | 0.014 |
| Yes | 537 (42.65) | 347 (42.01) | 190 (43.88) |  | 0.038 | 379 (44.17) | 191 (44.52) | 188 (43.82) |  | -0.014 |
| Renal disease, n (%) |  |  |  | 0.057 |  |  |  |  | 1.000 |  |
| No | 834 (66.24) | 532 (64.41) | 302 (69.75) |  | 0.116 | 598 (69.7) | 299 (69.70) | 299 (69.70) |  | 0.000 |
| Yes | 425 (33.76) | 294 (35.59) | 131 (30.25) |  | -0.116 | 260 (30.3) | 130 (30.30) | 130 (30.30) |  | 0.000 |
| Heart rate (beats/min) | 88.14 ± 16.11 | 87.57 ± 15.92 | 89.23 ± 16.44 | 0.081 | 0.101 | 89.38 ± 16.11 | 89.71 ± 15.81 | 89.06 ± 16.41 | 0.555 | -0.040 |
| MBP (mmHg) | 75.63 ± 10.13 | 75.63 ± 9.99 | 75.62 ± 10.39 | 0.991 | -0.001 | 75.67 ± 10.04 | 75.64 ± 9.68 | 75.70 ± 10.40 | 0.924 | 0.006 |
| SpO_2_ (%) | 96.46 ± 2.17 | 96.38 ± 2.20 | 96.61 ± 2.12 | 0.063 | 0.113 | 96.62 ± 2.07 | 96.64 ± 2.03 | 96.61 ± 2.12 | 0.831 | -0.014 |
| Glucose (mg/dL) | 137 (113, 172) | 136 (111, 170) | 140 (117, 173) | 0.149 | 0.044 | 138 (115, 171) | 137 (112, 170) | 140 (117, 173) | 0.269 | 0.033 |
| Hemoglobin (g/L) | 9.9 (8.4, 11.4) | 9.8 (8.3, 11.4) | 10.0 (8.6, 11.5) | 0.120 | 0.101 | 10.0 (8.5, 11.6) | 10.0 (8.4, 11.8) | 10.0 (8.5, 11.5) | 0.958 | 0.018 |
| Platelets (K/uL) | 204 (141, 281) | 199 (137, 278.75) | 210 (152, 286) | 0.020 | 0.118 | 209 (146, 285) | 208 (141, 285) | 210 (152, 285) | 0.626 | 0.023 |
| WBC (K/uL) | 9.9 (7.1, 13.5) | 9.7 (6.8, 13.2) | 10.2 (7.6, 14.3) | 0.009 | 0.111 | 10.5 (7.6, 14.2) | 10.7 (7.6, 14.2) | 10.1 (7.6, 14.1) | 0.488 | -0.076 |
| BUN (mg/dL) | 25 (16, 41) | 26 (17, 41) | 24 (16, 40) | 0.204 | -0.058 | 25 (16, 41) | 26 (18, 41) | 24 (16, 40) | 0.068 | -0.074 |
| Sodium (mEq/L) | 137 (133, 140) | 137 (134, 140) | 137 (133, 140) | 0.424 | -0.038 | 137 (133, 140) | 137 (133, 140) | 137 (133, 140) | 0.752 | 0.039 |
| Potassium (mEq/L) | 3.9 (3.5, 4.3) | 3.9 (3.5, 4.3) | 3.9 (3.5, 4.4) | 0.339 | 0.073 | 3.9 (3.5, 4.3) | 3.9 (3.5, 4.3) | 3.9 (3.5, 4.3) | 0.527 | 0.048 |
| GCS | 15 (13, 15) | 15 (13, 15) | 15 (13, 15) | 0.904 | 0.014 | 15 (13, 15) | 15 (13, 15) | 15 (13, 15) | 0.813 | 0.014 |
| SOFA | 5 (3, 8) | 5 (3, 8) | 5 (3, 7) | 0.173 | -0.090 | 5 (3, 8) | 5 (3, 8) | 5 (3, 7) | 0.818 | -0.035 |
| Charlson | 6 (4, 8) | 6 (4, 8) | 6 (4, 8) | 0.105 | -0.111 | 6 (4, 8) | 6 (4, 8) | 6 (4, 8) | 0.458 | -0.065 |
| SIRS score | 3 (2, 3) | 3 (2, 3) | 3 (2, 4) | <.001 | 0.258 | 3 (2, 4) | 3 (2, 4) | 3 (2, 4) | 0.684 | -0.032 |
| MV, n (%) |  |  |  | 0.897 |  |  |  |  | 0.881 |  |
| No | 881 (69.98) | 579 (70.10) | 302 (69.75) |  | -0.008 | 604 (70.4) | 303 (70.63) | 301 (70.16) |  | -0.010 |
| Yes | 378 (30.02) | 247 (29.90) | 131 (30.25) |  | 0.008 | 254 (29.6) | 126 (29.37) | 128 (29.84) |  | 0.010 |
| CRRT, n (%) |  |  |  | 0.135 |  |  |  |  | 1.000 |  |
| No | 1,238 (98.33) | 809 (97.94) | 429 (99.08) |  | 0.119 | 850 (99.07) | 425 (99.07) | 425 (99.07) |  | 0.000 |
| Yes | 21 (1.67) | 17 (2.06) | 4 (0.92) |  | -0.119 | 8 (0.93) | 4 (0.93) | 4 (0.93) |  | 0.000 |

Abbreviations: PSM, propensity score matching; SMD, standardized mean difference; MBP, mean blood pressure; SpO_2_, blood oxygen saturation; WBC, white blood cell count; BUN, blood urea nitrogen; GCS, Glasgow Coma Scale; SOFA, Sequential Organ Failure Assessment; CCI, Charlson Comorbidity Index; SIRS, Systemic Inflammatory Response Syndrome; MV, mechanical ventilation; CRRT, continuous renal replacement therapy.


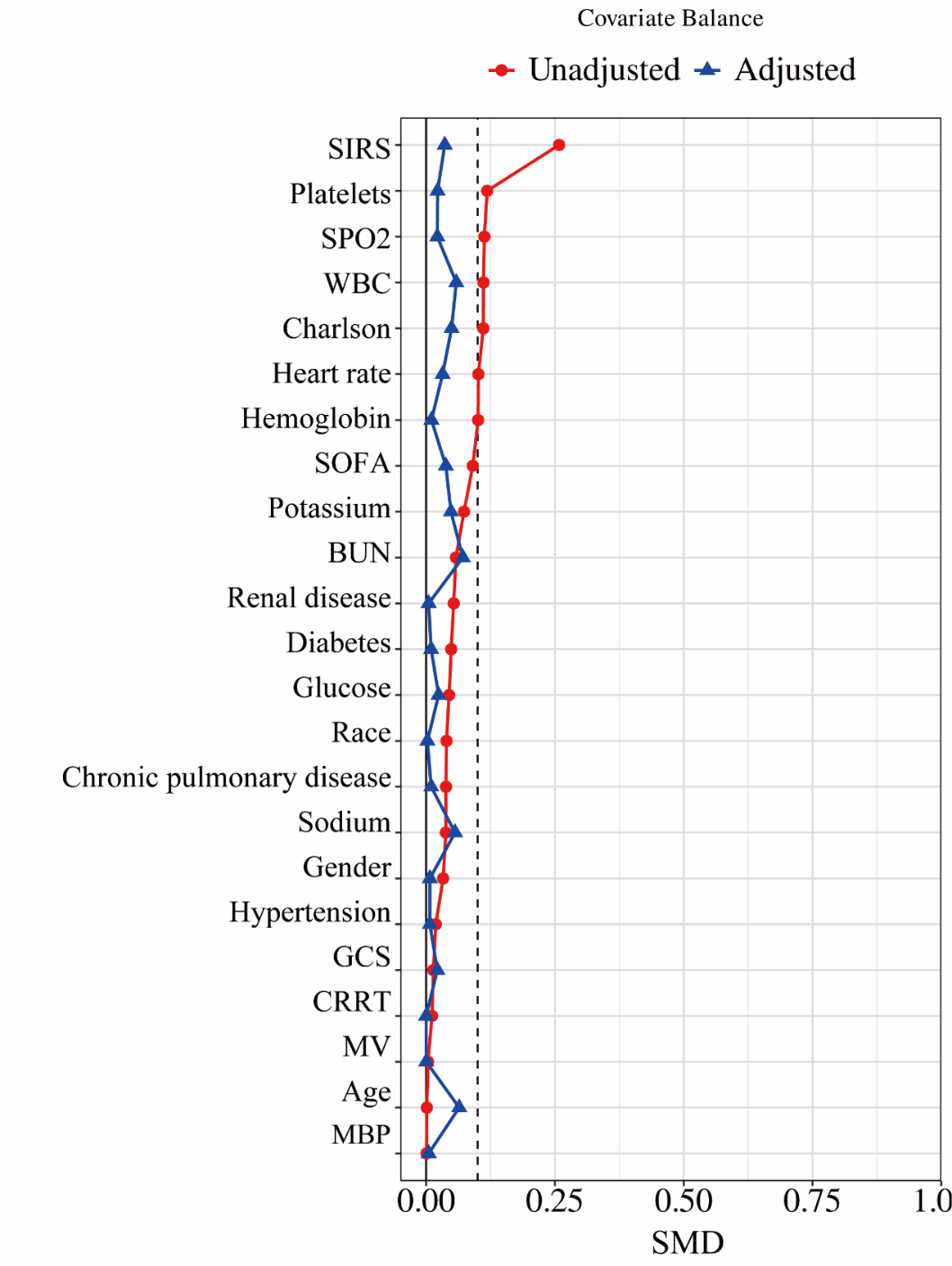


Figure S1. Standardized mean difference of variables before and after PSM. PSM, propensity score matching; SMD, standardized mean difference; MBP, mean blood pressure; SpO2, blood oxygen saturation; WBC, white blood cell count; BUN, blood urea nitrogen; GCS, Glasgow Coma Scale; SOFA, Sequential Organ Failure Assessment; CCI, Charlson Comorbidity Index; SIRS, Systemic Inflammatory Response Syndrome; MV, mechanical ventilation; CRRT, continuous renal replacement therapy.
